# *DES*ign of Sustainable *One-Pot* Chemoenzymatic Organic Transformations in *Deep Eutectic Solvents* for the Synthesis of 1,2-Disubstituted Aromatic Olefins

**DOI:** 10.3389/fchem.2020.00139

**Published:** 2020-03-06

**Authors:** Nicolás Ríos-Lombardía, María Jesús Rodríguez-Álvarez, Francisco Morís, Robert Kourist, Natalia Comino, Fernando López-Gallego, Javier González-Sabín, Joaquín García-Álvarez

**Affiliations:** ^1^EntreChem SL, Vivero Ciencias de la Salud, Santo Domingo de Guzmán, Oviedo, Spain; ^2^Laboratorio de Compuestos Organometálicos y Catálisis (Unidad Asociada al CSIC), Departamento de Química Orgánica e Inorgánica (IUQOEM), Centro de Innovación en Química Avanzada (ORFEO-CINQA), Universidad de Oviedo, Oviedo, Spain; ^3^Institute of Chemistry, Organic & Bioorganic Chemistry, NAWI Graz, BioTechMed Graz, University of Graz, Graz, Austria; ^4^Heterogeneous Biocatalysis Laboratory, CIC biomaGUNE, Donostia-San Sebastian, Spain

**Keywords:** *Deep Eutectic Solvents*, chemoenzymatic, metal-catalysis, biocatalysis, metathesis, phenolic acid decarboxylase, Heck reaction

## Abstract

The self-assembly of styrene-type olefins into the corresponding stilbenes was conveniently performed in the *Deep Eutectic Solvent* (*DES*) mixture 1*ChCl*/2*Gly* under air and in the absence of hazardous organic co-solvents using a one-pot chemo-biocatalytic route. Here, an enzymatic decarboxylation of *p*-hydroxycinnamic acids sequentially followed by a ruthenium-catalyzed metathesis of olefins has been investigated in *DES*. Moreover, and to extend the design of chemoenzymatic processes in *DESs*, we also coupled the aforementioned enzymatic decarboxylation reaction to now concomitant Pd-catalyzed Heck-type C-C coupling to produce biaryl derivatives under environmentally friendly reaction conditions.

## Introduction

Following the basis of the *Sustainable Development* (United Nations, 2015), and trying to confront both the current diminution of crude oil resources and the dramatic environmental difficulties connected with its use, the *Chemical Community* is making great efforts to employ bio-based feedstock as starting materials instead of non-renewable fossil supplies, therefore attempting to accomplish some of the essential *Principles* of *Green Chemistry* (Anastas and Warner, 1998; Matlack, 2001; Lancaster, 2002; Poliakoff et al., 2002; Sheldon et al., 2007). Nowadays, one of the major challenges of *Sustainable Chemistry* is replacing the use of organic solvents in chemical reactions since their production mainly relies on petrochemical manufacturing (Constable et al., 2007). In this sense, it is estimated that around 90% of the mass balance of chemical processes is associated with the solvents employed not only in the synthetic methodologies but also in the isolation and purification steps of the target products (Clark and Tavener, 2007; Jessop, 2011). Traditionally, organic chemists have employed volatile, hazardous and non-renewable petroleum-based *Volatile Organic Compounds* (*VOCs*) as reaction media taking advantage of the so-called “*positive solvent effect*” (Reichardt and Welton, 2010). In the search of maximizing this “*positive solvent effect”* while deviating from the previously commented traditional hazardous *VOC* solvents, chemists have endeavored to research new greener and non-conventional reaction media (Anastas, 2010), which should be easily available, biodegradable, non-toxic, biorenewable, and safe for both humans and the environment (Moity et al., 2012). In this context and during the last decade, a new family of sustainable reaction media, the so-called *Deep Eutectic Solvents* (*DESs*) (Abbott et al., 2003, 2004) has attracted the attention of many research groups worldwide. As consequence, these neoteric solvents have been broadly applied in chemistry as sustainable reaction media for: (*i*) polar organometallic chemistry (Mallardo et al., 2014; Vidal et al., 2014, 2016; Sassone et al., 2015; García-Álvarez et al., 2018; Rodríguez-Álvarez et al., 2018; Ghinato et al., 2019); (*ii*) metal catalyzed organic reactions (García-Álvarez, 2015; Vidal and García-Álvarez, 2019); (*iii*) biocatalysis (Gotor-Fernández and Paul, 2018); (*iv*) traditional organic synthesis (Liu et al., 2015; Alonso et al., 2016); (*v*) metal extraction and electrochemistry (Smith et al., 2014; Millia et al., 2018); and (*v*) polymer science (Carriazo et al., 2012; del Monte et al., 2014; Mota-Morales et al., 2018; Quirós-Montes et al., 2019; Roda et al., 2019; Sánchez-Condado et al., 2019). These sustainable eutectic solvents can be obtained just by mixing (without any further steps of purification or isolation) two molecules capable of forming a complex intermolecular network based on hydrogen-bond interactions. One molecule must be a HBA (hydrogen-bond-acceptor), whereas the other must act as hydrogen-bond-donor (HBD) (García-Álvarez, 2019). In this field, one of the most commonly used HBA for the synthesis of *DESs* is choline chloride (*ChCl*, [(*CH*_3_)_3_NCH_2_CH_2_OH]Cl), which is a safe and non-toxic (vitamin B_4_, RDA 550 mg) quaternary ammonium salt produced at multi-ton scale and employed as additive for feeding chicken (cost: 2€/Kg) (Blusztajn, 1998). In combination with different sustainable HBD [like glycerol, saccharides, urea or bio-based organic acid (i.e., lactic acid)], *ChCl* is able to form liquid eutectic mixtures with tunable physicochemical properties.

**Scheme 1:**
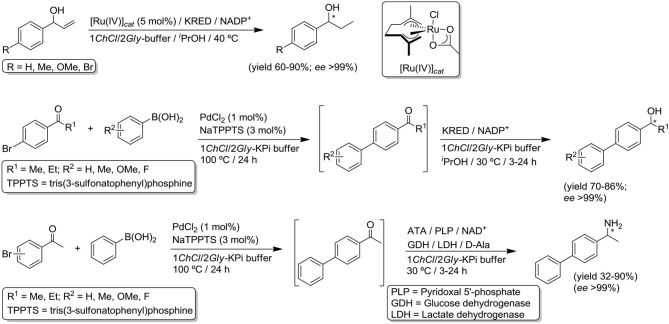
Previous reported examples on chemoenzymatic cascades in *DESs*.

On the other hand, during the last decade organic chemists have tried to design cleaner and more efficient one-pot multistep processes as alternative routes to classical step-by-step processes with the concomitant: (*i*) minimization of the time consumption and chemical waste; (*ii*) simplification of the practical aspects; and (*iii*) possibility to cope with sensitive reaction intermediates (no isolation of transiently-formed unstable products is needed; Hayashi, 2016). However, these one-pot multistep processes typically rely on the employment of the same synthetic organic tool (metal-, bio-, or organo-catalyzed reactions) throughout the transformation, while the corresponding hybrid counterparts combining different catalytic disciplines are still very scarce. In this sense, transition-metal-catalyzed reactions (van Leeuwen, 2004; Hartwig, 2010) represent an excellent platform to produce prochiral intermediates that enzymes can subsequently convert into high-added-value enantiopure compounds (Sheldon and Pererira, 2017). This chemoenzymatic approach has been fruitfully employed in traditional and hazardous organic solvents^1^ due to the intrinsic shortcomings (hydrolysis, oxidations) that transition-metal complexes usually suffered in water. However, the recent advances on the field of organometallic catalysis in water (Dixneuf and Cadierno, 2013) opened the door to its possible coupling with biotransformations in aqueous systems after overcoming other concomitant drawbacks related with: (*i*) reciprocal poisoning of catalysts; (*ii*) degradation because of additives, co-factors or co-solvents; (*iii*) incompatibility of reaction conditions; and (*iv*) undesired side-reactions (Gröger and Hummel, 2014; Bornscheuer, 2015; Schmidt et al., 2018). Despite these issues, current studies are boosting the aqueous combination of well-established metal-catalyzed organic reactions (Pd, Cu, or Ru-catalyzed processes) with enzyme-mediated aminations, reductions, halogenations, or decarboxylation processes (Gröger and Hummel, 2014; Bornscheuer, 2015; Ríos-Lombardía et al., 2018; Schmidt et al., 2018). Interestingly, the combination of transition metals and enzymes in the above-mentioned *DESs* has been barely noticed, despite the beneficial effect of these neoteric solvents in both metal-catalyzed transformations (García-Álvarez, 2015; Vidal and García-Álvarez, 2019) and enzymatic processes (Gotor-Fernández and Paul, 2018) is now well-established. In this sense, some of us have reported the pioneering combinations in *ChCl*-based eutectic solvents of the ruthenium(IV)-catalyzed isomerization of allylic alcohols with bioreductions (Cicco et al., 2018); or the assemble of Pd-catalyzed Suzuki C-C coupling with bioreductions (Ketoreductases, KRED; Paris et al., 2018) and bioaminations (transaminases, ATA) (Paris et al., 2019; see Scheme 1).

**Scheme 2:**
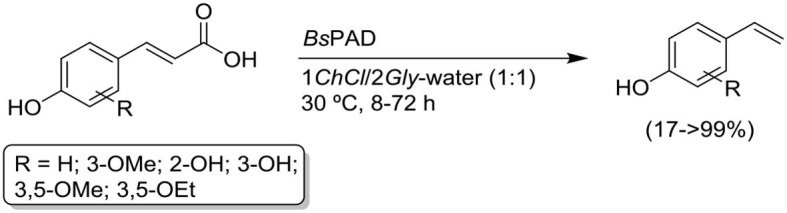
*Bs*PAD-catalyzed decarboxylation of *p*-hydroxycinnamic acids in the 1*ChCl*/2*Gly*-water medium.

Based on the excellent catalytic activity of Phenolic Acid Decarboxylase from *Bacillus subtilis* (*Bs*PAD) in the decarboxylation of *p*-hydroxycinnamic acids in *DESs* (see Scheme 2; Schweiger et al., 2019), we herein present the unprecedented one-pot combination of the selective production of *p*-hydroxystyrenes catalyzed by a *Bs*PAD in the eutectic mixture choline chloride/glycerol (1*ChCl*/2*Gly*) with two well-established metal-catalyzed organic processes that employ styrene-type precursors, like: (*i*) the Grubbs-II catalyzed metathesis of olefins (an organic reactions which remained unreported in eutectic mixtures); and (*ii*) the Pd-catalyzed Heck-type C-C coupling reaction^2^^,^^3^.

## Results and Discussion

### Ruthenium Catalyzed (Grubbs-II) Metathesis of Styrene-Type Olefins in *Deep Eutectic Solvents* (*DESs*)

Ru-catalyzed olefin metathesis is considered as one of the most efficient and reliable synthetic utensils within the organic synthetic toolbox for the selective formation of new C-C bonds, thus allowing the direct access to organic compounds that would require a larger number of conventional steps for their synthesis (Grubbs, 2003; Hoveyda and Zhugralin, 2007; Grela, 2014). In this sense, and while olefin metathesis in organic and hazardous *VOC* solvents is nowadays ubiquitous [and even in aqueous media (Lipshutz and Ghorai, 2008; Burtscher and Grela, 2009; Tomasek and Schatz, 2013)], the possibility to carry out ruthenium-catalyzed metathesis of olefins in biorenewable eutectic mixtures has got not precedents, as far as we are aware. Accordingly, and trying to finish with this discontinuity in the employment of *DESs* in transition-metal-catalyzed organic transformations, our experimental work started with the study of the archetypical self-assembly metathesis of styrene (**1a**) as a model reaction (see Table 1) by employing the Grubbs-II complex (4 mol%) in the eutectic mixture 1*ChCl*/2*Gly*, at 50°C and in the presence of air (entry 1, Table 1). After 20 h of reaction under the aforesaid mild reaction conditions, the desired stilbene (**2a**) was selectively obtained in almost quantitative yield (95%). For comparison, water (the prototypical example of green solvent) was studied under the same reaction conditions (4 mol% of Grubbs-II at 50°C), giving rise to lower yields of **2a** (87%, entry 2, Table 1) after longer reaction times (24 h), thus disclosing a new example of an accelerated organic reaction in *DESs* (García-Álvarez, 2015; Vidal and García-Álvarez, 2019). A similar reduction of the yield of the target **2a** was also observed when the catalyst loading was reduced by half (i.e., 2 mol%, entry 3, Table 1). After this first parametric study, we extended our investigations to other *ChCl*-based *DES* containing different hydrogen-bond-donors [HBD, urea (entry 4) or lactic acid (*Lac*, entry 5)], finding in both cases a similar decrease of the catalytic activity of the Grubbs-II catalyst (60–61%). Taking into consideration the experimental fact that uncovers 1*ChCl*/2*Gly* as the best reaction media, we investigated if the presence of the quaternary ammonium salt *ChCl* was necessary to achieve high conversions on the final stilbene (**2a**). Thus, the self-assembly metathesis of **1a** upon the optimized reaction conditions (50°C, 4 mol% of Grubbs-II and under air) was studied but employing pure glycerol as solvent (entry 6, Table 1). As expected from our previous experience on metal-catalyzed processes in *ChCl*-based *DESs* (García-Álvarez, 2015; Vidal and García-Álvarez, 2019), the reaction in the absence of *ChCl* (pure glycerol) also produced **2a** but in lower yield (71%) after longer reaction time (24 h), thus revealing the positive effect of non-molecular *ChCl*-based *DESs* when compared with their separated components.

**Table 1:** Parameterization studies on the Ru-catalyzed self-assembly metathesis of styrenes (**1a–f**) in different *Deep Eutectic Solvents* (*DESs*)^a^.

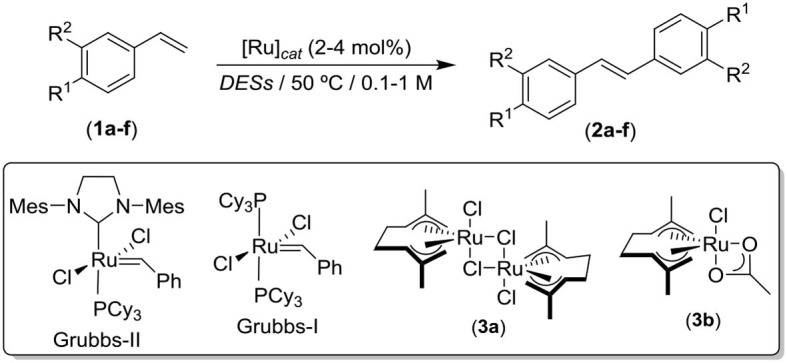									
**Entry**	**Substr**.	**R**^**1**^	**R**^**2**^	**Solvent**	**Catalyst**	**[Ru] (mol%)**	**Conc**.	***t*** **(h)**	***c*** **(%)^b^**
1	**1a**	H	H	1*ChCl*/2*Gly*	Grubbs-II	4	1 M	20	95^c^
2	**1a**	H	H	H_2_O	Grubbs-II	4	1 M	24	87
3	**1a**	H	H	1*ChCl*/2*Gly*	Grubbs-II	2	1 M	24	76
4	**1a**	H	H	1*ChCl*/2Urea	Grubbs-II	4	1 M	20	60
5	**1a**	H	H	1*ChCl*/2*Lac*	Grubbs-II	4	1 M	20	61
6	**1a**	H	H	*Gly*	Grubbs-II	4	1 M	24	71
7	**1a**	H	H	1*ChCl*/2*Gly*	Grubbs-I	4	1 M	24	15
8	**1a**	H	H	1*ChCl*/2*Gly*	**3a**	4	1 M	24	5
9	**1a**	H	H	1*ChCl*/2*Gly*	**3b**	4	1 M	24	2
10	**1a**	H	H	1*ChCl*/2*Gly*	Grubbs-II	4	0.1 M	24	14
11	**1b**	OMe	H	1*ChCl*/2*Gly*	Grubbs-II	4	1 M	24	83
12	**1c**	F	H	1*ChCl*/2*Gly*	Grubbs-II	4	1 M	24	21
13	**1d**	Cl	H	1*ChCl*/2*Gly*	Grubbs-II	4	1 M	24	17
14	**1a**	H	H	1*ChCl*/2*Gly*	–	4	1 M	24	0
15	**1e**	OH	H	1*ChCl*/2*Gly*	Grubbs-II	4	1 M	24	15
16	**1e**	OH	H	1*ChCl*/2*Gly*	Grubbs-II	4	0.5 M	24	12
17	**1e**	OH	H	*DES*:H_2_O (1:1)^d^	Grubbs-II	4	0.5 M	24	12
18	**1f**	OH	OMe	*DES*:H_2_O (1:1)^d^	Grubbs-II	4	0.5 M	24	18

a *General conditions: reactions performed under air at 50°C with 0.5 mmol of substrate and 0.5 mL of the desired solvent. For more information, see Supplementary Material*.

b *Determined by GC*.

c *91% isolated yield of **2a***.

d *1ChCl/2Gly:H_2_O 1:1*.

Next, we focus our attention on the activity of other ruthenium catalysts for the self-assembly metathesis of **1a** in 1*ChCl*/2*Gly* (entries 7–9). The Grubbs-I complex was tested as catalyst, discovering a dramatic decrease in the catalytic activity under the optimized reaction conditions as only 15% of **2a** was obtained after 24 h (entry 7, Table 1). Taking into account the high activity exhibited for the bis(allyl)-Ru(IV) complexes **3a,b** in the isomerization of allylic alcohols in *DESs*, these complexes were also tested in the metathesis of **1a** in the eutectic mixture 1*ChCl*/2*Gly*. Unfortunately, both complexes were almost inactive in such transformation (entries 8–9, Table 1). For completeness, the parameterization study included the effect of the substrate concentration in the course of the catalytic reaction. As a result, a decrease from 1 M of **1a** (entry 1) to 0.1 M (entry 10) produced a remarkable decrease in the final conversion (14%).

With the optimized reaction conditions in hand, the scope of the reaction was extended to other styrene-like olefins finding that electron donating groups (MeO, entry 11) are tolerated in the process (83%) while the presence of electron withdrawing groups (F, entry 12; Cl, entry 13) results in concomitant decrease of the activity of the Grubbs-II catalyst. Likewise, the presence of the Ru catalyst was verified as mandatory for the reaction to take place (entry 14, Table 1). Bearing in mind the planned cascade and the fact that *Bs*PAD decarboxylates exclusively *p*-hydroxycinnamic acids^4^, *p*-hydroxystyrene and 4-hydroxy-3-methoxystyrene (**1e–f**, the tentative products resulting from the biotransformation), were subjected to the metathesis reaction. Unfortunately, the *p*-hydroxyl group within **1e** exerted a strong negative effect and the measured conversion was exclusively of 15% (entry 15). Further attempts at 0.5 M and using a medium consisting of *DES*:H_2_O 1:1 led to similar outcome (entries 16–17). Analogously, the disubstituted **1f** styrene led to the stilbene-type derivative **2f** in 18%. At this point, and bearing in mind the results obtained in entries 11–18 (Table 1), it is important to highlight that we observed experimentally a direct relationship between the solubility of the styrenes **1a–f** in the eutectic mixture 1*ChCl*/2*Gly* and the reaction yield. In this sense, and for the case of styrene (**1a**) and *p*-methoxy-styrene (**1b**), which are totally insoluble in the eutectic mixture 1*ChCl*/2*Gly*, the catalytic reaction takes place under heterogeneous conditions and best reaction yields were observed (95 and 83%, respectively). However, we observed a dramatic decrease in the yield of the reaction (12–21%) when partially soluble (**1c–d**) or soluble (**1e–f**) styrenes were employed^5^^,^ ^6^. Similarly, some of us have previously reported an analogous scenario when using organolithium reagents (RLi) in the heterogeneous anionic polymerization of the aforementioned styrenes **1a-d** when employing polar eutectic mixtures as reaction media (Sánchez-Condado et al., 2019).

As stated above, the inhibition of the catalytic activity of transition-metal complexes in the presence of enzymes, co-factors or buffers is a well-known phenomenon in one-pot chemoenzymatic processes (Gröger and Hummel, 2014; Bornscheuer, 2015; Schmidt et al., 2018). Accordingly, we designed two experiments aimed at verifying the compatibility of the co-factor free *Bs*PAD-catalyzed decarboxylation process with the Grubbs-II catalyzed self-assembly metathesis of styrenes in *DESs*^7^. Initially, the catalytic activity of the Grubbs-II catalyst in the metathesis of **1a** was evaluated in the medium employed in the enzymatic decarboxylation, namely a mixture 1*ChCl*/2*Gly*:H_2_O 1:1 (see Scheme 2). Similarly, in a second experiment the enzyme was also added to the reaction media 1*ChCl*/2*Gly*:H_2_O containing the Grubbs-II catalyst. Pleasantly, both tests showed that the metallic complex suffers virtually no erosion of its catalytic activity (96% of **2a**) in the reaction conditions needed for the starting enzymatic step of the planned cascade process.

### Chemoenzymatic Combination of *Bs*PAD-Catalyzed Decarboxylation of *p*-Hydroxycinnamic Acids With the Ru-Catalyzed (Grubbs-II) Metathesis of Styrene-Type Olefins in *DESs*

**Scheme 3:**
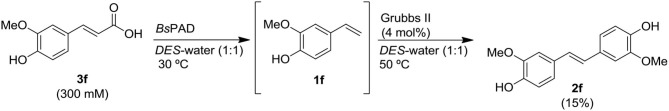
One-pot sequential enzymatic decarboxylation (*Bs*PAD)/metal-catalyzed metathesis (Grubbs-II) of *p*-hydroxycinnamic acid (**3f**) in the mixture 1*ChCl*/2*Gly*-water.

Encouraged by the previous parametrization that suggest the possibility to trigger the self-assembly metathesis of *p*-hydroxystyrenes employing directly the reaction media coming from the enzymatic decarboxylation of *p*-hydroxycinnamic acids, we designed the chemoenzymatic coupled process in a 1*ChCl*/2*Gly*-water medium (see Scheme 3). According to conditions optimized previously (Schweiger et al., 2019), the *Bs*PAD-catalyzed decarboxylation of **3f** was performed at 300 mM substrate concentration and 30°C in 1*ChCl*/2*Gly*-water. Once the biotransformation was completed, the Grubbs-II catalyst was then added and the reaction mixture stirred for 24 h at 50°C. As expected, the conversion in the metathesis step was very low and the isolated overall yield of **2f** for the two-steps process resulted in 15%. Hence, our studies revealed that the stepwise enzymatic decarboxylation/metal-catalyzed metathesis was hampered by the intrinsic limitations of the metallic step, not by the incompatibility between single reactions. At least, it can be taken as a proof of concept of the viability of this chemoenzymatic process in *DES*-water media at the expense of developing more efficient catalysts for the metathesis of *p*-hydroxystyrenes.

### Chemoenzymatic Combination of the *Bs*PAD-Catalyzed Decarboxylation of *p*-Hydroxycinnamic Acids With the Pd-Catalyzed Heck Coupling in *DESs*

**Scheme 4:**
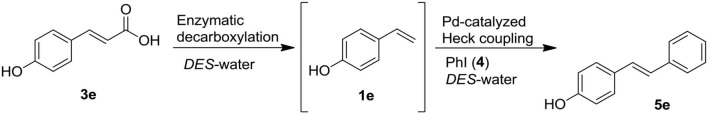
One-pot sequential enzymatic decarboxylation (*Bs*PAD)/Pd(PPh_3_)_4_-catalyzed Heck coupling of *p*-hydroxycinnamic acid (**3e**) with PhI (**4**) in *DES*-water mixtures.

Seeing as the above unsatisfactory results, we turned our attention on other metal-catalyzed transformations reported in eutectic mixtures, like the case of the Pd-catalyzed Heck-type C-C coupling^2, 3^. In fact, such chemoenzymatic process, namely the tandem enzymatic decarboxylation/Heck coupling has just been reported in continuous flow in *DES*-water to tackle incompatibility of catalysts and solubility issues (Grabner et al., 2020). Accordingly, we planned to get more insight about this system by setting up the counterpart process in batch conditions (Scheme 4). Following the reported conditions in the flow system, we essayed the Heck coupling between *p*-hydroxystyrene (**1e**) and iodobenzene (**4**) in 1*ChCl*/2*Gly*:H_2_O 1:1 but employing the commercially available catalyst Pd(PPh_3_)_4_. The use of EtOH as co-solvent (25% v/v) proved to be critical for the outcome of the reaction. Working at 100°C and 60 mM of **4**, the conversion reached a value >90% with an isolated yield of 65% on (*E*)-4-hydroxystilbene (**5e**) after 8 h. In fact, due to the strong reaction conditions, the yield was limited by the formation of the isomeric byproduct *p*-hydroxy-1,1-diphenylethylene at a percentage close to 10%. On the other hand, the inhibition studies unveiled that by adding exogenous *Bs*PAD the Pd catalyst is deactivated (c = 0%) which precludes the chemoenzymatic process without additional settings.

In a first attempt to remove the biocatalyst for the tentative second step of the cascade, a *Bs*PAD-catalyzed decarboxylation of **3e** was conducted at 120 mM and 30°C in 1*ChCl*/2*Gly*:H_2_O 1:1 during 2 h. Once the biotransformation was accomplished (99%), the reaction mixture was subjected to centrifugation at 13,000 rpm during 5 min. The insolubles were discarded and the supernatant diluted (up to 60 mM of PhI, the limiting reactant) and treated with the reagents for the Heck coupling. As a result, the overall yield for the two-steps process was exclusively of 15%, which suggests that *Bs*PAD partially remained in the supernatant. Hence, two strategies were planned to avoid the contact between catalysts: (*i*) immobilization of *Bs*PAD on a solid support; or (*ii*) employment of aqueous micellar solutions which enable to isolate metal catalyst and enzyme from each other. For more information, see Supplementary Material.

**Figure 1:**
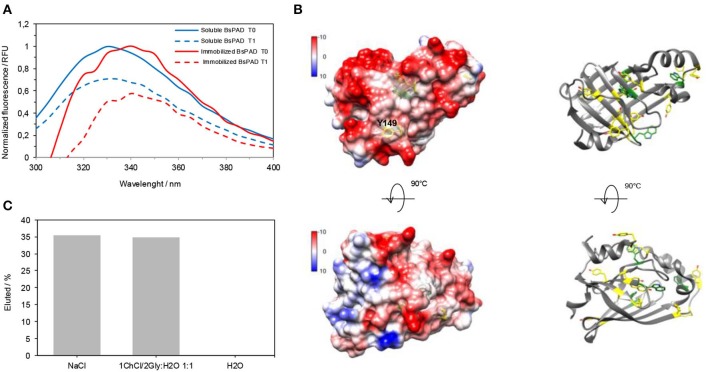
**(A)** Total intrinsic fluorescence of soluble and immobilized *Bs*PAD using an excitation wavelength of 280 nm. T0 (solid lines) correspond to spectra of the non-incubated enzymes, while T1 (dashed lines) do to enzyme incubated for 1 h at 45°C. Data were normalized assigning a value of 1 to the highest fluorescence intensity value for each sample non-thermally incubated (sample T0s). **(B)** Electrostatic surface representation of *Bs*PAD (PDB ID: 2P8G). Tryptophan and tyrosine residues are colored in green and yellow, respectively. Images were made with Chimera software. **(C)** Percentage of protein eluted from the carrier after an incubation of 1 h with 1 M NaCl, a solution of (1*ChCl*/2*Gly*:H_2_O 1:1) and H_2_O.

With regards to the first approach, we conceived a solid support for *Bs*PAD which fitted the requirements for the chemoenzymatic cascade; preserving the catalytic activity of the enzyme and avoiding the enzyme lixiviation to the reaction medium based on *DES*-water mixtures. With these premises, we selected the commercially available carrier EP-403S (pore size: 40–60 nm and particle size: 100–300 μm). We further activated such carrier with tertiary amine groups (EP-TEA) to promote the enzyme immobilization through reversible but strong ionic interactions as reported for other industrially relevant enzymes as ketoreductases (Benitez-Mateos et al., 2017). 100% of the offered *Bs*PAD was immobilized on the carrier although the specific activity of the enzyme was reduced to 51% upon the immobilization (Table 2). Noteworthy, the immobilized *Bs*PAD was significantly more active than other decarboxylases immobilized on pre-existing carriers through both reversible and irreversible chemistries (Aßmann et al., 2017). When we performed fluorescence studies, we observed that the intrinsic Trp-fluorescence intensity of *Bs*PAD decreased and its maximum emission wavelength was red-shifted when the enzyme was immobilized (Figure 1A). That fluorescence decay suggests that the immobilization induced some conformational distortions on *Bs*PAD structure that would explain its lower recovered enzyme. Inspecting the X-ray structure of *Bs*PAD, we found a region clearly enriched in Asp and Glu, which makes us to postulate that region as the one through the immobilization takes place. Moreover, in that acidic region, we find two main tyrosines that may be quenched to some extent upon the immobilization (Figures 1A,B). Hence, we suggest that the lower catalytic efficiency of this heterogeneous biocatalyst may rely on some structural distortion at the interface between that acidic region of the enzyme and the surface of acrylic porous beads.

**Table 2:** Heterogeneous biocatalyst characterization.

**Enzyme**	**Support**	**Ψ %**	**A_**i**_ (U/g)**	**Load (mg/g)**	**A_**R**_ (U/g)**	**A_**R**_ %**	**Ae_**i**_ (U/mg)**
*Bs*PAD	EP-TEA	100	3,09	1	1,58	51,11	1,58

*Immobilization parameters of BsPAD in ECGP/403-5/TEA support. Ψ%, immobilization yield; A_i_, immobilized activity; A_R_, restored activity; Ae_i_, expressed activity of immobilized enzyme*.

We named the resulting heterogeneous biocatalysts as PAD@EC-TEA, which despite presenting lower specific activity than the soluble enzyme, exhibited an excellent decarboxylation efficiency toward **3e** both in water and 1*ChCl*/2*Gly*:H_2_O 1:1. In particular, working at 200 mM of **3e** and a comparable loading of biocatalyst, the reaction was complete in 2 h (*c* > 99%). Remarkably, PAD@EC-TEA could be efficiently recycled up to four times in H_2_O meanwhile the heterogeneous biocatalyst completely lost its activity after just one cycle in the *DES* mixture. This fact was due to the high concentration of choline needed to form the *DES* (3 M) that partially released *Bs*PAD from the aminated carrier (Figure 1C). In fact, the *DES* was as efficient as 1 M NaCl breaking the electrostatic interactions between the proteins and the positively charged carrier, and therefore eluting *Bs*PAD to the reaction crude. Then, two decarboxylation reactions of **3e** were run at 120 mM in 1*ChCl*/2*Gly*:H_2_O 1:1 and water. After verifying complete conversion of the cinnamate by HPLC, the reaction mixtures were filtered off to remove PAD@EC-TEA and subjected to further Heck coupling (60 mM). Using the selected *DES* mixture, no product was detected as expected since choline partially eluted the *Bs*PAD to the reaction crude (Figure 1C), thus that soluble enzyme inhibited the Pd catalyst, precluding the formation of the C-C bond (Table 3, entry 2). On the contrary, running the decarboxylation in water, the enzyme remained bound to the carrier and consequently the metal-catalyzed reaction rendered **5e** in 60% overall yield (Table 3, entry 3). Therefore, this two-step one-pot sequential chemoenzymatic process successfully worked up when firstly performing the *Bs*PAD-driven decarboxylation in water, separation the heterogeneous biocatalysts by simple vacuum filtration and finally diluting the reaction by 2-fold with the *DES* and adding the Pd-catalyst. Using this synthetic sequence, we are able to achieve high yields of the desired product and recycle the immobilized *Bs*PAD.

**Table 3:** Strategies for the one-pot sequential enzymatic decarboxylation/Pd-catalyzed Heck coupling of *p*-hydroxycinnamic acid (**3e**) in non-conventional media^a^.

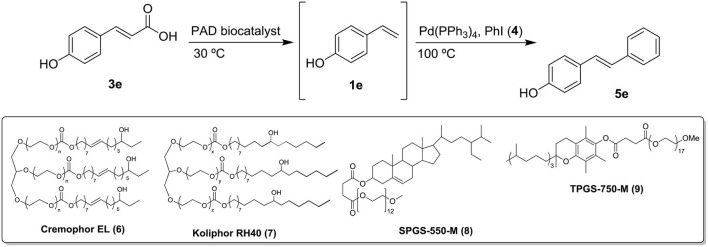									
			**Enzymatic decarboxylation**				**Pd-catalyzed Heck coupling**		
**Entry**	**Substr**.	**Biocatalyst**	**Medium**	**[3e](mM)**	***c*** **(%)^b^**	**Work-up^c^**	**Medium**	**[1e] (mM)**	**Yield (%)^e^**
1	**3e**	PAD WT	*DES*:H_2_O 1:1	120	>99	A	*DES*:H_2_O:EtOH	60	15
2	**3e**	PAD-TEA	*DES*:H_2_O 1:1	120	>99	A	*DES*:H_2_O:EtOH	60	-
3	**3e**	PAD-TEA	H_2_O	120	>99	B	*DES*:H_2_O:EtOH	60	60
4	**3e**	PAD WT	H_2_O (2 wt.% **6**)	120	>99	C	H_2_O:EtOH^d^	60	60
5	**3e**	PAD WT	H_2_O (2 wt.% **7**)	120	>99	C	H_2_O:EtOH^d^	60	55
6	**3e**	PAD WT	H_2_O (2 wt.% **8**)	120	95	C	H_2_O:EtOH^d^	60	25
7	**3e**	PAD WT	H_2_O (2 wt.% **9**)	120	>99	C	H_2_O:EtOH^d^	60	20
8	**3e**	PAD-TEA	H_2_O (2 wt.% **6**)	120	55	B	H_2_O:EtOH^d^	60	<5
9	**3e**	PAD-TEA	H_2_O (2 wt.% **7**)	120	40	B	H_2_O:EtOH^d^	60	<5
10	**3e**	PAD WT	H_2_O (2 wt.% **6**)	200	>99	C	H_2_O:EtOH^d^	60	62
11	**3e**	PAD WT	H_2_O (2 wt.% **6**)	200	>99	C	H_2_O:EtOH^d^	100	70

a *General conditions: The enzymatic decarboxylation was performed under air at 30°C during 2 h. The Heck coupling was performed at 100°C during 8 h*.

b *Determined by HPLC*.

c *Procedure A: centrifugation and removal of insolubles. Procedure B: removal of biocatalyst by filtration through a cartridge. Procedure C: dilution in the reaction medium of the second step without further treatment*.

d *Water contains 2 wt.% of the corresponding surfactant*.

e *Overall yield for the two-steps procedure after silica gel chromatography*.

Very recently, designer surfactants based on aqueous micellar solutions have been demonstrated as valuable reaction media for chemoenzymatic cascades (Cortes-Clerget et al., 2019). In particular, Lipshutz et al. developed several one-pot two-step combinations of metal-catalyzed reactions with a subsequent bioreduction. In the so-called micellar catalysis, the biocatalyst remains in the aqueous solution, while the organic solvents host the metal-catalyst and acts as reservoir for substrate and product. On the other hand, the solubilizing properties of the surfactant Cremophor® enabled to enhance the substrate concentration of poorly-water soluble substrates in a two-enzyme cascade reaction (Correia Cordeiro et al., 2019). With these precedents, the *Bs*PAD-catalyzed decarboxylation of **3e** was essayed at 200 mM in a medium consisting of water and 2 wt.% of different surfactants (above their reported critical micelle concentration)^8^. As deduced from Table 3, the reaction proceeded smoothly in such a low percentage of the four solubilizers tested (c > 90% entries 4–7). Then, the corresponding reaction mixtures were supplemented with an aqueous solution (2 wt.% solubilizer) containing the Pd catalyst, EtOH, PhI, and K_2_CO_3_ and heated at 100°C during 8 h. The media containing Cremophor EL (**6**) and Kolliphor RH40 (**7**) led to good yields (55–60%, entries 4–5), in contrast to those containing both SPGS-550-M (**8**) and TPGS-750-M (**9**) (20–25%, entries 6–7)^9^. Likewise, the immobilized biocatalyst (*Bs*PAD-TEA) was also tested in the best micellar solutions (those containing **6** and **7**). Unfortunately, such media resulted in incomplete decarboxylation of **3e** (*c* < 55%, entries 8–9) which led to very low overall yields after the metal-catalyzed step (*c* < 5%). Finally, the effect of substrate concentration on the two-steps protocol was studied. The enzymatic decarboxylation of **3e** took place warmly at 200 mM in the micellar solution containing **6** (entry 10). Further dilution to 60 mM in PhI for the Heck coupling (as in previous experiments) led to comparable isolated yield (62%). Gratefully, the attempt to enhance the second step to 100 mM resulted in an improved overall yield (70%, entry 11).

## Conclusions

The application of non-conventional media such as *Deep Eutectic Solvents* (*DESs*) or aqueous micellar solutions as a practical solution to set up two chemoenzymatic cascades has been studied. In particular, the *Bs*PAD-catalyzed decarboxylation of *p*-hydroxycinnamic acids was coupled alternatively with two metal-catalyzed processes such as the metathesis of olefins and a Heck C-C coupling reaction. In the first process, both catalysts were compatible and the low overall yield was due to the ineffective activity of the Grubbs-II catalyst toward *p*-hydroxystyrenes in such eutectic mixtures. With regards to the second, the employment of an immobilized biocatalyst on the one hand or micellar solutions on the other was enough to confine the enzyme and avoid its inhibitory activity on the Pd catalyst, the target biaryl derivatives being obtained in moderate to good yields. Despite some limitations remained challenging in the processes reported herein, these examples contribute to expand the available toolbox for developing chemoenzymatic cascades. Future efforts must be led to improve the immobilization of *Bs*PAD on solid materials to achieve irreversibly bound enzymes that do not interfere with the metal-catalyzed step and tolerate *DES* mixtures to assemble one-pot concurrent systems.

## Data Availability Statement

The raw data supporting the conclusions of this article will be made available by the authors, without undue reservation, to any qualified researcher.

## Author Contributions

NR-L and MR-Á carried out chemical synthesis and characterization of the products. FM contributed to the manuscript revision and writing. RK developed and supplied the enzyme *Bacillus subtilis* (*Bs*PAD). NC and FL-G have designed and characterized the supported enzyme (*Bs*PAD-TEA). JG-S and JG-Á designed and managed the study and also carried out manuscript writing. All the authors approved the publication of the work.

### Conflict of Interest

NR-L, FM, and JG-S were employed by the company Entrechem S.L. The remaining authors declare that the research was conducted in the absence of any commercial or financial relationships that could be construed as a potential conflict of interest.

## Footnotes

^1^For selected recent examples, see: (Lohr and Marks, 2015; Verho and Bäckvall, 2015; El-Sepelgy et al., 2016; Palo-Nieto et al., 2016; Yang et al., 2016; Görbe et al., 2017).

^2^For previous examples that described palladium-catalyzed Heck-type C-C coupling processes in *DESs*, see: (Illgen and König, 2009; Marset et al., 2017; Saavedra et al., 2019).

^3^At this point, we would like to highlight that Ramón *et al*. have nicely studied (by DFT calculations and NMR techniques) the possible catalytically active Pd-species present in the Heck reaction in eutectic mixtures (see Marset et al., 2017).

^4^The mechanism of the *Bs*PAD-catalyzed decarboxylation of *p*-hydroxycinnamic acids starts by deprotonation of the *p*-hydroxy group and proceeds *via* a quinone methide intermediate, which then leads to the cleavage of CO_2_. For the specific case of *Deep Eutectic Solvents*, it has been reported that: “*in view of the observed differing substrate preference dependent on the applied solvent, it is suggested that solvent effects play a crucial role in our studied reaction systems besides classical steric and electronic properties of the substrates*.” For more information, see: (Rodríguez et al., 2010).

^5^These experimental facts suggest that the catalytic reaction takes place “*on the eutectic mixture*” and not “*in the eutectic mixture*”. This “*on DES*” concept is closely related with the “*on water*” concept previously coined by K. B. Sharpless. These “*on water*” (or “*on DES*”) reactions are thought to occur at the organic/liquid water (or eutectic mixture) interface with water (or *DES*) insoluble reactants. For leading references, dealing with the “*on water*” concept, see: (Narayan et al., 2005; Chanda and Fokin, 2009; Butler and Coyne, 2015, 2016).

^6^This mechanistic proposal indicates that the eutectic-phobic interactions in between both the insoluble styrenes and the catalyst permit an intimal contact between both reactants, thus increasing the overall observed catalytic rate of the process. This mechanistic effect is now well-established and has been previously observed by different research groups worldwide in the study of Grubbs-catalyzed “*on water*” metathesis processes. For outstanding articles and book chapters that reported previous examples of this mechanistic effect in Grubbs-catalyzed metathesis reactions see: (Gułajski et al., 2008; Diallo et al., 2010; Grela et al., 2015).

^7^For previous examples dealing with the combination of Ru-catalyzed metathesis processes and different enzymatic transformations in aqueous media, see: (Tenbrink et al., 2011; Denard et al., 2014; Gómez Baraibar et al., 2016; Scalacci et al., 2017; Bojarra et al., 2018).

^8^The technical specifications for the commercially available Kolliphor RH40® and Cremophor EL® report a critical micelle concentration (CMC) of 0.03% w/w at 37°C and 0.02% w/w at 25°C, respectively. With regards to the recently designed surfactants TPGS-750-M and SPGS-550-M, they exhibited a CMC of *ca*. 10^−4^ M and 2% w/w was found to be useful for most reactions studied to date: (Lipshutz and Ghorai, 2012).

^9^The observed decrease in the catalytic activity when using TPG-750-M and SPGS-550M could be explained by bearing in mind the fact that their technical specifications assessed that: *reactions can be run between 4 and 60*°*C. Above 70*°*C, however, particle shapes become unpredictable; e.g., reorganization from spherical particles to bilayer arrays may occur, and so the quality of reactions run in these modified particles is not predictable*.
