# Supplementary material for: DESign of Sustainable One-Pot Chemoenzymatic Organic Transformations in Deep Eutectic Solvents for the Synthesis of 1,2-Disubstituted Aromatic Olefins
Source: Front Chem. 2020 Mar 6;8:139. doi: 10.3389/fchem.2020.00139 (PMC7067824; doi:10.3389/fchem.2020.00139)
Supplement: Supplementary file 1 [file Data_Sheet_1.docx]

Supplementary Material

*DES*ign of Sustainable *One-Pot* Chemoenzymatic Organic Transformations in *Deep Eutectic Solvents* for the Synthesis of 1,2-Disubstituted Aromatic Olefins

Nicolás Ríos-Lombardía^1^, María Jesús Rodríguez-Álvarez^2^, Francisco Morís^1^, Robert Kourist^3^, Natalia Comino,^4^ Fernando López-Gallego^4^, Javier González-Sabín^1*^ and Joaquín García-Álvarez^2*^

*** Correspondence:**

[jgsabin@entrechem.com](mailto:jgsabin@entrechem.com) (J.G.-S.)

[garciajoaquin@uniovi.es](mailto:garciajoaquin@uniovi.es) (J.G.-A.)

Table of Contents

1. General information (p. S2)
2. Experimental procedures (p. S5)
3. GC and HPLC analytical data (p.S8)
4. Copy of HPLC chromatograms for the one-pot processes (p. S10)
5. Copy of NMR spectra (p. S12)
6. Bibliography (p. S15)

**General information**

# *Reagents*

Styrene-type reagents and the components of the eutectic mixture were directly purchased from Sigma Aldrich. Tetrakis(triphenylphosphine)palladium(0) was purchased from Sigma Aldrich. Surfactants Cremophor EL, Koliphor RH40, SPGS-550-M and TPGS-750-M were purchased from Sigma Aldrich.

# *Ruthenium catalysts*

Grubbs-I and Grubbs-II catalysts were purchased directly from Sigma Aldrich. Bis(allyl)-ruthenium(IV) catalysts **3a-b** were synthesized as previously reported in the literature [1].

# *Enzymes*

**Expression of wild-type *Bs*PAD.** *E. coli* Bl21 (DE 3) cells harboring the plasmid of PAD was used to inoculate overnight cultures (5 mL LB-Kan), which were incubated at 37 °C and 130 rpm. The overnight culture was used to inoculate 200 mL TB-Kan medium. The cells were grown at 37  ºC and 130 rpm until OD_600_ of 0.5-0.7 was reached, before expression was started by induction with IPTG (0.1 mM). The cultures were incubated at 20 ºC and 120 rpm for 14 h and the cells were harvested by centrifugation (15 min, 4500 rpm, 4 °C). The resulting pellet was washed with 50 mM potassium phosphate buffer (pH 6) before storage at ‑20 °C.

**Preparation of cell-free extract and enzyme purification.** The pellet was resuspended (100 mg/mL) in either reaction buffer (50 mM potassium phosphate buffer, pH 6) or purification buffer (20 mM Tris-HCl, 300 mM NaCl, 5 mM imidazole, pH 7.4) and the cells were disrupted by sonication (Branson sonifier 250; 5 min, Duty Cycle 5, Output control 50%). After centrifugation (20 min, 11000 rpm, 4 °C) the supernatant was sterile-filtered. For His-tag purification, Ni Sepharose™ 6 Fast Flow (GE Healthcare) column material was used according to the manufacturer’s instructions. The purification process was monitored with SDS-PAGE. The buffer of the combined collected elution fractions was exchanged to 50 mM potassium phosphate buffer (pH 6) with PD-10 Desalting Columns (GE Healthcare) before the enzyme solution was concentrated in Vivaspin® 20 centrifugal concentrators (Sartorius, MWCO 10 kDa). The protein concentration was determined with a Pierce™ BCA Protein Assay Kit (ThermoFisher Scientific) and the purified enzyme was stored in 20% glycerol at ‑20  ºC.

*Preparation of immobilized biocatalyst*

***Activation of EP403-S with tertiary amine groups (EP-TEA).*** 10 g of EP403-5 beads were incubated with 100 mL of 1M 3-(diethylamino)ethanol at pH 10 for 2 hours under gentle stirring at room temperature. Then, the solid material was washed with an excess of water to remove the unreacted triethylamine. Afterwards, the beads were incubated with 0.5 M of sulfuric acid in order to hydrolyze the remaining epoxy groups that were not modified during the amination step. The resulting carrier contained 30 μmol of tertiary amines per gram of carrier.

***Bs*PAD immobilization on EP-TEA.** For *Bs*PAD immobilization 500 mg of EP-TEA carrier were incubated with 5 mL of *Bs*PAD solution at 0,1 mg mL^-1^ in 25 mM phosphate buffer pH 7 for 1 h at room temperature with orbital shacking. The immobilization course was monitored by measuring activity in the supernatant. The immobilization yield (Ψ) was calculated as follows [Eq. (1)]:

$$\text{Ψ }\left( \text{\%} \right)\text{= }\frac{{\text{A}_{\text{offered}} -\text{ A}}_{\text{supernant}}}{\text{A}_{\text{offered}}} \times100$$

in which A_offered_ is the activity (U/mL) of the soluble *Bs*PAD offered to the carrier while A_supernant_ is the activity (U/mL) that remains in the supernant after the immobilization time.

**Enzymatic assays.** The activity of *Bs*PAD was measured colorimetrically using *p*-nitrophenol (*p*NP) as pH indicator. This compound is colorless below pH 5.4 and yellow above 7.5 with a maximum of absorbance at 405 nm. The CO_2_ releasing promoted by *Bs*PAD activity induces an increase in the pH that turns the pH-indicator into a yellow colored compound that can be spectrophotometrically quantified. We calibrated the enzymatic assay to correlate the change of color with the CO_2_ concentration. The enzymatic assays were carried out in a Varioskan^®^ Flash (Thermo Scientific) on 96-well plates under orbital shacking. The reaction mixture containing 10 mM ferulic acid, 10% DMSO, 0,5 mM *p*NP in 25 mM sodium phosphate buffer was adjusted at pH 6. 20 µL of enzymatic solution or suspension in 25 mM sodium phosphate buffer pH 7 were incubated with 200 µL of reaction mixture at 25 °C. The *Bs*PAD activity was expressed in international units (U) defined as the amount of protein needed to neutralize 1 µmol of H^+^ per minute.

**Intrinsic fluorescence assays.** The intrinsic fluorescence of soluble and immobilized *Bs*PAD was measured exciting a suspension 1:3 (w:v) of the immobilized enzyme at 280 nm and recording the emission spectrum between 300-500 nm. The study was performed with 200 µL of soluble or immobilized *Bs*PAD (66,7 µg protein amount per assay) in 25 mM sodium phosphate buffer pH 7 using a Varioskan^®^ Flash (Thermo Scientific) and 96-well plates.

**Desorption assays.** 50 mg of immobilized *Bs*PAD were resuspended in 150 µL of 1M NaCl, 1*ChCl*/2*Gly*:H_2_O 1:1 and H_2_O, respectively. After 1 h incubation at room temperature with orbital shacking protein concentration in the supernatant was measured by Bradford’s assay [2].

*Preparation of DES*

*Deep Eutectic Solvent* *ChCl*-*Gly* (1:2 mol/mol), was prepared by gently heating under stirring at 60-80 ºC for 1 hour the corresponding individual components until a clear solution was obtained.

# *General methods*

^1^H-NMR spectra (CDCl_3_) were obtained using a Bruker DPX-300 (^1^H, 300.13 MHz) spectrometer using the δ scale (ppm) for chemical shifts. Calibration was made on the signal of the solvent (^1^H: CDCl_3_, 7.26). HPLC analyses to determine degree of conversion were carried out in an Agilent RR1200 HPLC system, using a reversed phase column (Zorbax Eclipse XDB-C18, RR, 18 μm, 4.6 x 50 mm, Agilent). Gas chromatography (GC) analyses were performed on an Agilent Technologies 7820A chromatograph equipped with a HP-5 (30 m x 0.32 mm x 0.25 μm) column.

**Experimental procedures**

*Ruthenium-catalyzed self-assembly metathesis of styrenes* ***1a-d*** *in different Deep Eutectic Solvents (DESs)*

The desired styrenes **1a-d** (0.5 mmol) and 0.5 g of the required eutectic mixture were introduced into a reaction vial. Subsequently the ruthenium catalyst (2-4 mol%) was added and the obtained reaction was heated at 50 ºC. The course of the reaction was monitored by regular sampling and analysis by GC. The identity of the resulting stilbene-type products **2a-d** was confirmed by ^1^H-NMR analyses after comparison with reported spectra.

*BsPAD-catalyzed decarboxylation of p-hydroxycinnamic acid (****3e****) in DES-water mixtures*

In a 2.0 mL glass vial, **3e** (49 mg, 0.30 mmol) and lyophilized *Bs*PAD (16 mg) were added to 2.5 mL of 1*ChCl*/2*Gly*-water 1:1. The reaction was shaken at 250 rpm and 30 ºC for 2 h. To monitor the reaction, an aliquot was taken (10 µL) and diluted with MeOH (700 μL). After centrifugation (90 sec, 13000 rpm), the conversion was determined by HPLC. Once the biotransformation was complete, NH_4_Cl was added (1.0 mL) and the mixture extracted with EtOAc (2 x 2.0 mL). Then, the organic layers were separated by centrifugation (90 sec, 13000 rpm), combined and finally dried over Na_2_SO_4_. Further evaporation to dryness in a vacuum concentrator yielded pure **1e** (>95% yield).

*BsPAD-catalyzed decarboxylation of p-hydroxycinnamic acid (****3e****) in aqueous micellar solutions*

In a glass vial, **3e** (49 mg, 0.30 mmol) and lyophilized *Bs*PAD (16 mg) were added to 2.5 mL of an aqueous solution (2 wt% solubilizer). The reaction was shaken at 250 rpm and 30 ºC for 2 h. The reaction was monitored by HPLC as described above. Then, the resulting mixture was extracted with EtOAc (2 x 2.0 mL). Next, the organic layers were separated by centrifugation (90 sec, 13000 rpm), combined and finally dried over Na_2_SO_4_. Further evaporation to dryness in a vacuum concentrator yielded pure **1e** (>95% yield).

*Ru-catalyzed self-assembly metathesis of 4-hydroxy-3-methoxystyrene (****1f****) in DES-water mixtures*

To a solution of **1f** (75 mg, 0.50 mmol) in 1*ChCl*/2*Gly*-water 1:1 (0.5 mL), Grubbs-II catalyst was added (16 mg, 0.020 mmol) and the mixture stirred at 50 ºC during 24 h. After this time, the reaction mixture was cooled to room temperature and quenched by addition of saturated aqueous NH_4_Cl (1.0 mL). The mixture was extracted with ethyl acetate (2 × 2.0 mL) and the organic layers were separated, combined and dried over anhydrous Na_2_SO_4_ to provide the crude product. Further filtration by flash chromatography (silica gel 60 Å, hexane:ethyl acetate mixtures) yielded **2f** (15%).

*Pd-catalyzed Heck coupling of 4-vinylphenol (****1e****) and iodobenzene (****4****) in DES-water mixtures*

To a solution of **1e** (0.30 mmol) in 3.3 mL of *DES*:H_2_O:EtOH (1:1:0.6) were consecutively added potassium carbonate (0.30 mmol), iodobenzene (0.20 mmol) and Pd(PPh_3_)_4_ (2 mg, 1% mol) and the resulting mixture heated at 100 ºC during 8 h. Then the reaction mixture was cooled to room temperature and quenched by addition of saturated aqueous NH_4_Cl (2.0 mL) and 1N HCl (1.0 mL). The mixture was extracted with ethyl acetate (2 × 3.0 mL) and the organic layers were separated, combined and dried over anhydrous Na_2_SO_4_ to provide the crude product. Further filtration by flash chromatography (silica gel 60 Å, hexane:ethyl acetate mixtures) yielded (*E*)-4-hydroxystilbene (**5e**, 65%).

*One-pot BsPAD-catalyzed decarboxylation-ruthenium-catalyzed metathesis of p-hydroxycinnamic acid (****3e****) in DES-water mixtures*

In a glass vial, **3f** (73 mg, 0.375 mmol) and lyophilized *Bs*PAD (24 mg) were added to 1.25 mL of 1*ChCl*/2*Gly*-water 1:1 and the mixture shaken at 30 ºC for 2 h. Then, Grubbs-II catalyst was added (13 mg, 0.015 mmol) and the mixture stirred at 50 ºC during 24 h. After this time, the reaction mixture was cooled to room temperature and quenched by addition of saturated aqueous NH_4_Cl (2.0 mL). The mixture was extracted with ethyl acetate (2 × 2.0 mL) and the organic layers were separated, combined and dried over anhydrous Na_2_SO_4_ to provide the crude product. Further filtration by flash chromatography (silica gel 60 Å, hexane:ethyl acetate mixtures) yielded **2f** (15%).

*One-pot BsPAD-catalyzed decarboxylation-Pd-catalyzed Heck coupling of p-hydroxycinnamic acid (****3e****) in aqueous micellar solutions*

In a round-bottom flask, **3e** (82 mg, 0.50 mmol) and lyophilized *Bs*PAD (27 mg) were added to 2.5 mL of an aqueous solution (2 wt% of **6**) and the mixture shaken at 30 ºC for 2 h. Then, were consecutively added potassium carbonate (0.33 mmol), iodobenzene (0.33 mmol), Pd(PPh_3_)_4_ (4 mg, 1% mol), EtOH (0.8 mL) and the resulting mixture heated at 100 ºC during 8 h. Next, the reaction mixture was cooled to room temperature and quenched by addition of saturated aqueous NH_4_Cl (2.0 mL) and 1N HCl (1.0 mL). The mixture was extracted with ethyl acetate (2 × 5.0 mL) and the organic layers were separated, combined and dried over anhydrous Na_2_SO_4_ to provide the crude product. Further filtration by flash chromatography (silica gel 60 Å, hexane:ethyl acetate mixtures) yielded (*E*)-4-hydroxystilbene (**5e**, 70%).

**GC and HPLC analytical data**

**3.1. GC analytical data**

*Analytical data for the determination of the degree of conversion (c) of the metathesis reaction*

Gas chromatography (GC) analyses were performed on a Hewlett Packard 6890 Series II chromatograph using the column Beta Dex (120 m 0.25 mm 0.25μm). The employed program started at an initial temperature of 60 ºC (4 min) and then a slope of 20 ºC/min until to reach a final temperature of 310 ºC.

**Table S1.** GC analysis data.

| **Substrate** | **Retention time (min)** | |
| --- | --- | --- |
|  | Substrate | Product |
| styrene (**1a**) | **1a** (2.547) | **2a** (10.737) |
| 4-methoxystyrene (**1b**) | **1b** (6.835) | **2b** (14.502) |
| 4-fluorostyrene (**1c**) | **1c** (2.691) | **2c** (10.740) |
| 4-chlorostyrene (**1d**) | **1d** (5.960) | **2d** (14.504) |

**3.2.** **HPLC analytical data**

*Analytical data for the determination of the degree of conversion (c) of the enzymatic decarboxylation and the Pd-catalyzed Heck coupling*

HPLC analyses were carried out in an Agilent chromatographic system, using a reversed phase column (Zorbax Eclipse XDB-C18, RR, 1.8 μm, 4.6 x 50 mm, Agilent) and acetonitrile (MeCN) and 0.1% trifluoroacetic acid (TFA) in water as solvents. Samples were eluted with three linear gradients from 10% to 60% MeCN during 5.70 min, followed by another from 60% to 100% MeCN during 0.5 min and a third gradient from 100% to 10% MeCN during 1.90 min, at flow rate of 2 mL/min. Detection and spectral characterization of peaks were performed with a diode array detector and ChemStation Rev.B.03.01 software (Agilent).

**Table S2**. HPLC analytical data

| **Compound** | ***t*_R_ (min)** | **Wavelength (nm)** |
| --- | --- | --- |
| **3e** | 1.7 | 228 |
| **1e** | 3.4 | 228 |
| **4** | 5.8 | 210 |
| **5e** | 5.5 | 210 |
| **6e** | 6.6 | 210 |
| **3f** | 2.0 | 210 |
| **1f** | 3.6 | 210 |
| **2f** | 5.4 | 210 |

**Copy of HPLC chromatograms for the one-pot processes**

4.1 One-pot enzymatic decarboxylation-metathesis of **3f** in *DES*-water mixtures (Scheme 3)

********

4.2 One-pot enzymatic decarboxylation-Heck coupling of **3e** in aqueous micellar solutions (Table 2, entry 11)

**Copy of NMR spectra**

**Figure S1.** ^1^H-NMR full chart for **2a** in CDCl_3_ (300 MHz)

**Figure S2.** ^1^H-NMR full chart for **2b** in CDCl_3_ (300 MHz)

**Figure S3.** ^1^H-NMR full chart for **2c** in CDCl_3_ (300 MHz)

**Figure S4.** ^1^H-NMR full chart for **2d** in CDCl_3_ (300 MHz)


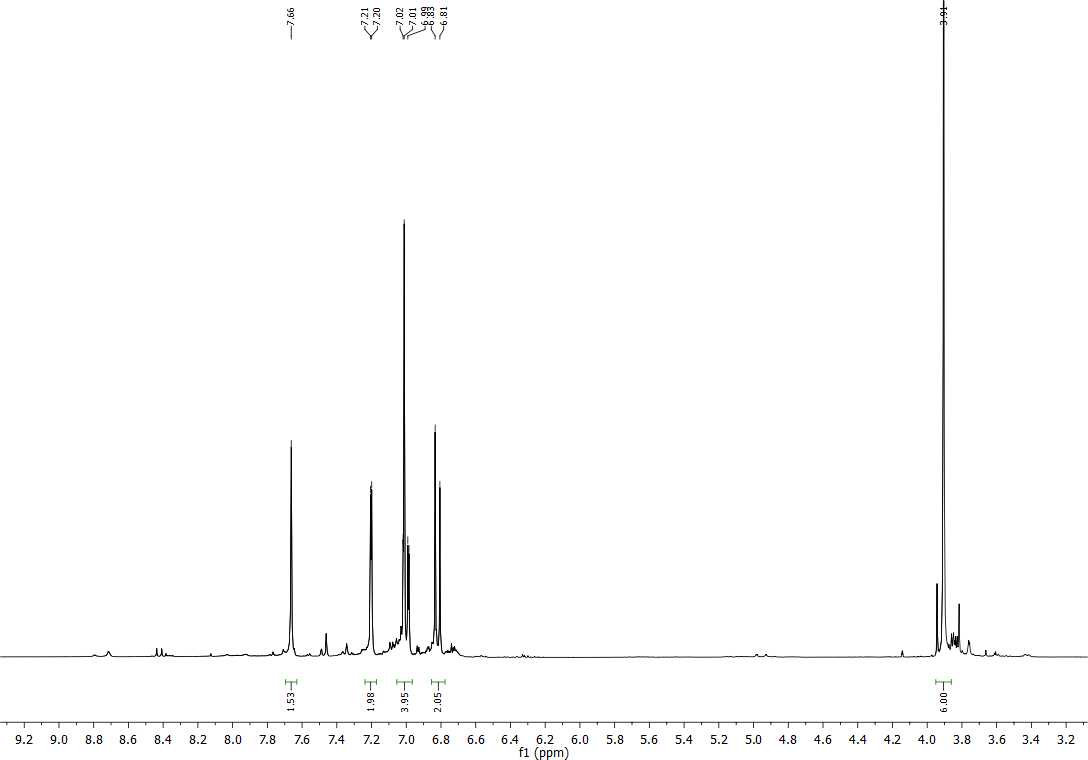


**Figure S5.** ^1^H-NMR full chart for **2f** in CDCl_3_ (300 MHz)

**
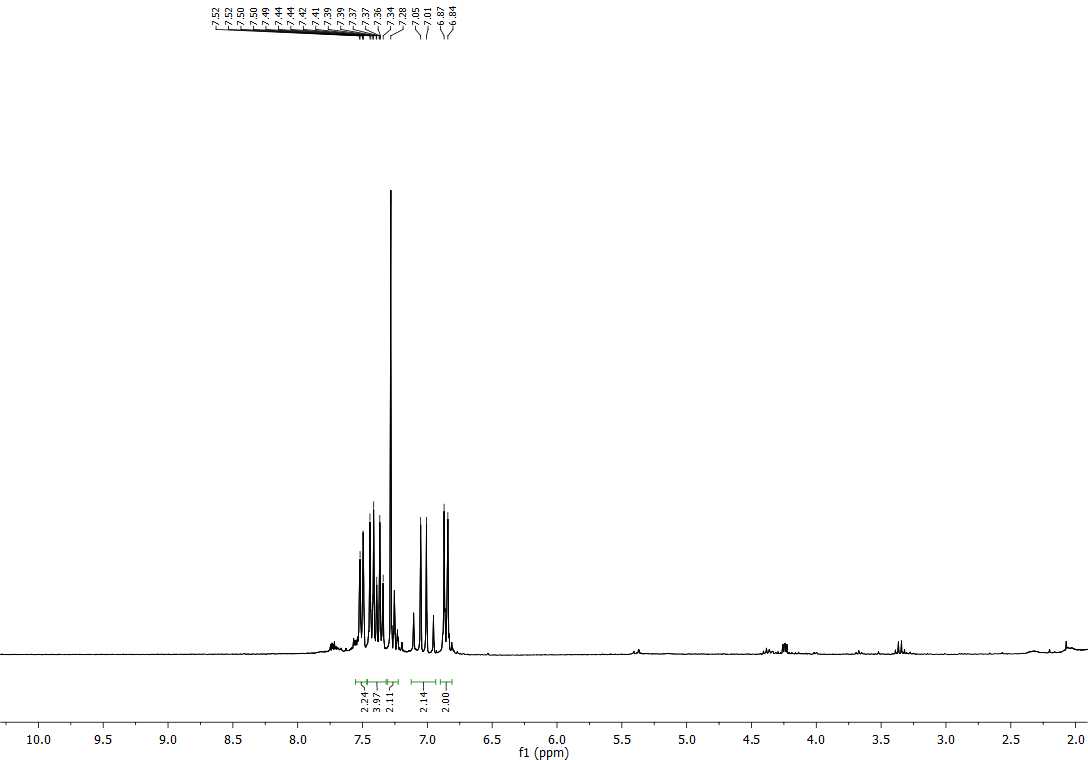
**

**Figure S6.** ^1^H-NMR full chart for **5e** in CDCl_3_ (300 MHz)

**Bibliography**

[1] J. García-Álvarez, J. Gimeno, F. J. Súarez, *Organometallics*, **2011**, *30*, 2893.

[2] M. M. Bradford, *Anal. Biochem.,* **1976**, *72*, 248.
